# Intake of Energy Drinks Before and During Pregnancy and Adverse Pregnancy Outcomes

**DOI:** 10.1001/jamanetworkopen.2023.44023

**Published:** 2023-11-20

**Authors:** Ming Ding, Andre O. Markon, Olivia E. Jones-Dominic, Alexandra C. Purdue-Smithe, Janet W. Rich-Edwards, Beverly J. Wolpert, Jorge E. Chavarro

**Affiliations:** 1 Department of Emergency Medicine, University of North Carolina, Chapel Hill; 2 Department of Nutrition, Harvard T.H. Chan School of Public Health, Boston, Massachusetts; 3 Center for Food Safety and Applied Nutrition, Office of Analytics and Outreach, US Food and Drug Administration, College Park, Maryland; 4 Division of Women’s Health, Brigham and Women’s Hospital and Harvard Medical School, Boston, Massachusetts; 5 Channing Division of Network Medicine, Brigham and Women’s Hospital and Harvard Medical School, Boston, Massachusetts; 6 Department of Epidemiology, Harvard T.H. Chan School of Public Health, Boston, Massachusetts

## Abstract

**Importance:**

Consumption of energy drinks has increased drastically in recent years, particularly among young people. It is unknown whether intake of energy drinks is associated with health during pregnancy.

**Objective:**

To examine associations of energy drink intake before and during pregnancy with risk of adverse pregnancy outcomes (APOs).

**Design, Setting, and Participants:**

This prospective cohort study included data from women enrolled in the Nurses’ Health Study 3 (NHS3) between June 1, 2010, and September 27, 2021, and the Growing Up Today Study (GUTS) who reported 1 or more singleton pregnancy from January 1, 2011, to June 1, 2019. Data were analyzed from October 1, 2021, to September 28, 2023.

**Exposure:**

Intake of energy drinks, assessed by food frequency questionnaire.

**Main Outcomes and Measures:**

The main outcomes were self-reported APOs, including pregnancy loss, gestational diabetes, gestational hypertension, preeclampsia, or preterm birth, and a composite APO, defined as development of any of the APOs. Risk of APOs was compared between consumers and nonconsumers of energy drinks.

**Results:**

This study included 7304 pregnancies in 4736 participants with information on prepregnancy energy drink intake and 4559 pregnancies in 4559 participants with information on energy drink intake during pregnancy. There were 1691 GUTS participants (mean [SD] age, 25.7 [2.9] years) and 3045 NHS3 participants (mean [SD] age, 30.2 [4.1] years). At baseline, 230 GUTS participants (14%) and 283 NHS3 participants (9%) reported any intake of energy drinks. While no associations were found for pregnancy loss (odds ratio [OR], 0.89; 95% CI, 0.71-1.11), preterm birth (OR, 1.07; 95% CI, 0.71-1.61), gestational diabetes (OR, 0.89; 95% CI, 0.58-1.35), preeclampsia (OR, 0.73; 95% CI, 0.41-1.30), or the composite APO (OR, 1.05; 95% CI, 0.87-1.26), prepregnancy energy drink use was associated with a higher risk of gestational hypertension (OR, 1.60; 95% CI, 1.12-2.29). A significant interaction was found between age and energy drink intake in relation to hypertensive disorders (*P* = .02 for interaction for gestational hypertension; *P* = .04 for interaction for any hypertensive disorders), with stronger associations for participants above the median age. No associations of energy drink intake during pregnancy with any of the APOs were found in NHS3 (eg, any APO: OR, 0.86; 95% CI, 0.41-1.79).

**Conclusions and Relevance:**

In this study, energy drink intake before pregnancy was associated with an elevated risk of gestational hypertension. Given the low prevalence of energy drink intake and low consumption levels among users, the results should be interpreted cautiously.

## Key Points

**Question:**

Is intake of energy drinks before and during pregnancy associated with adverse pregnancy outcomes?

**Findings:**

In this cohort study of 7304 pregnancies in 4736 participants and 4559 pregnancies in 4559 participants with information on energy drink intake before and during pregnancy, respectively, energy drink intake before pregnancy was associated with an elevated risk of gestational hypertension. No associations were found for pregnancy loss, preterm birth, gestational diabetes, preeclampsia, or a composite of adverse pregnancy outcomes.

**Meaning:**

Use of energy drinks before pregnancy may be associated with a higher risk of gestational hypertension.

## Teaser

This cohort study uses data from the Nurses’ Health Study and the Growing Up Today Study to assess the association of energy drink intake before and during pregnancy with risk of adverse pregnancy outcomes.

## Introduction

Energy drinks are beverages that are advertised to increase alertness, energy levels, and physical performance, often containing caffeine, guarana, taurine, and/or other substances. Sales of energy drinks have substantially increased by 240% in the US and worldwide since 1987,^1^ reaching over $9.7 billion in US sales in 2015.^2^ Energy drinks are popular especially among young adults; the National Health and Nutrition Examination Survey found that 5.5% of adults aged 20 to 39 years consume energy drinks.^3^ For college students, surveys conducted among hundreds of participants showed that 16% of participants were frequent consumers of energy drinks.^4,5^

Marketing campaigns of energy drinks have often targeted young people. However, there are concerns about the safety of energy drinks. Acute intake of energy drinks has been shown to be associated with increased systolic (SBP) and diastolic blood pressure (DBP).^6^ Cross-sectional studies found that energy drink intake was positively associated with mental health symptoms, such as stress, anxiety, and depression,^7^ and caffeine-related health complaints, including insomnia, headache, irritation, tiredness, and fatigue.^8,9^ Case reports showed that energy drinks may be associated with nausea, vomiting, hepatic and kidney damage, and even death.^10,11^ Given that the majority of energy drink consumers are young adults of reproductive age,^5^ including individuals who may become pregnant, it is of high importance to examine associations of energy drink intake with adverse pregnancy outcomes (APOs). However, few studies have addressed this issue, which is likely due to the lack of available longitudinal data allowing the prospective assessment of the association between energy drink intake and pregnancy-related outcomes. In this study, we examined the potential association between energy drink intake before and during pregnancy with APOs in 2 ongoing prospective cohort studies actively observing women of reproductive age: the Nurses’ Health Study 3 (NHS3) and the Growing Up Today Study (GUTS).

## Methods

### Study Population

This cohort study included data from GUTS and NHS3 and was conducted according to the guidelines of the Declaration of Helsinki.^12^ The institutional review board of the Brigham and Women’s Hospital approved NHS3 and GUTS. Completion of questionnaires was considered implied informed consent. This study followed the Strengthening the Reporting of Observational Studies in Epidemiology (STROBE) reporting guideline.

The GUTS started in 1996 when invitations to complete a baseline questionnaire were sent to 25 000 children and adolescents aged 9 to 14 years whose mothers consented to their recruitment; 16 882 children and adolescents (9033 girls) returned a completed questionnaire (GUTS1). The study was expanded in 2004 with the enrollment of an additional 10 923 children and adolescents (6002 girls) aged 9 to 14 years (GUTS2). Participants have been followed-up with self-administered follow-up questionnaires administered every 1 to 3 years since then. Participants reported energy drink intake frequency in 2011 and in a 2015 follow-up questionnaire and reported pregnancies and APOs on the 2019 questionnaire (eFigure 1 in Supplement 1). We included data from January 1, 2011, to June 1, 2019.

The NHS3 is an ongoing, internet-based cohort study of nurses that started enrolling participants in 2010.^13^ Registered nurses, licensed practical or vocational nurses, or nursing students born on or after January 1, 1965, living in the US and Canada are eligible for participation. At enrollment, participants complete questionnaires regarding lifestyle, demographic, occupational, and medical characteristics. Follow-up questionnaires are sent approximately every 6 months. In each follow-up questionnaire, participants are asked about their pregnancy status. Women reporting that they are currently pregnant on any questionnaire are invited to answer additional questions about their pregnancies, with questionnaires sent between 20 and 25 weeks’ gestation and 8 weeks after the estimated due date. Given that not all eligible participants complete the mid-pregnancy and postpregnancy questionnaires, all participants are also asked to update their information on all pregnancies taking place after baseline, including outcome, duration, and diagnosis of major complications for each pregnancy, in a supplementary reproductive questionnaire introduced in 2017 and repeated approximately every 30 months thereafter. As of September 2021, 35 555 female nurses had answered the second study questionnaire, which included questions about energy drink intake (eFigure 2 in Supplement 1). We included data from June 1, 2010, and September 27, 2021.

### Exposure Assessment

Participants in both cohorts completed a validated 131-item food frequency questionnaire (FFQ).^14,15^ To assess energy drink consumption, participants were asked to report how often, on average, they consumed energy drinks during the previous year using 9 categories ranging from “never or less than once per month” to “6 or more times per day.” In GUTS, participants were asked about energy drink intake in 2 questions regarding intake of regular energy drinks and intake of sugar-free, low-calorie, or low-carbohydrate energy drinks. In NHS3, participants reported total energy drink intake in a single question, with the same response options as in GUTS. In addition, participants in NHS3 reported on their diet during pregnancy by completing an FFQ between 20 and 25 weeks’ gestation. This questionnaire had the same food list, including the same question about energy drinks, but the reference time window for reporting was the first 20 weeks of pregnancy.

To maintain a strictly prospective analysis of prepregnancy energy drink intake and APOs, we first used information on the date when the pregnancy ended and the gestational age at pregnancy end to identify and exclude from the study pregnancies that started before diet assessment. We excluded pregnancies in women who received a diagnosis of type 2 diabetes or hypertension before pregnancy. We further excluded pregnancies with missing information on energy drink intake and twin and multiple pregnancies. In GUTS, we used the most recent dietary information preceding pregnancy to define prepregnancy energy drink intake. In other words, we used energy drink assessment from 2011 for pregnancies that occurred between the return of the 2011 and 2015 questionnaires and energy drink assessment in 2015 for pregnancies that occurred after the return of 2015 questionnaire. If energy drink intake was missing in 2015, we carried forward the information from the 2011 questionnaire. In NHS3, we used dietary information assessed in module 2 to define prepregnancy energy drink intake, and intake reported at 20 to 25 weeks’ gestation to define energy drink intake during pregnancy.

### Outcome Assessment

In GUTS, women reported their current and previous pregnancies in the 2019 follow-up questionnaire, including pregnancy end date, pregnancy length, pregnancy outcomes, and pregnancy complications. In NHS3, women reported their most recent pregnancies in mid- and postpregnancy questionnaires and all previous pregnancies in the supplementary reproductive questionnaire. We considered individual APOs, including pregnancy loss, gestational diabetes, gestational hypertension, preeclampsia, or preterm birth, as well as a composite APO variable (≥1 of these individual APOs). Pregnancy loss was defined as a fetal loss taking place before 20 weeks’ gestation, and preterm birth was defined as a pregnancy ending before 37 weeks’ gestation. Gestational diabetes, gestational hypertension, and preeclampsia were self-reported for each pregnancy. Self-reported pregnancy outcome and gestational age have been shown to be validly reported.^16^ The sensitivity of reporting a pregnancy loss among those that truly occurred is estimated to be around 75%.^17,18^ The positive predictive value of self-reported preeclampsia was 89% compared with medical records.^19,20^

### Covariate Assessment

Information on potential confounding variables, including age, educational level, self-reported race and ethnicity, smoking status, physical activity, dietary quality, total energy intake, multivitamin supplement use, and shift work (NHS3 only), was assessed using questionnaires at baseline. Body mass index (BMI; calculated as weight in kilograms divided by height in meters squared) was determined from self-reported weight and height. Race and ethnicity categories included Asian, Black, Hispanic, White, and other (American Indian and Hawaiian or Pacific Islander). The racial and ethnic minority groups were combined into 1 category in the analysis because of the small number of participants in each group. Participants were asked about the amount of time that they spent per week, on average, in each of the following physical activities: walking, jogging, running, bicycling, playing tennis, other aerobic exercise, lower-intensity exercise, and other vigorous activities. The amount of total reported physical activity was calculated as energy expenditure in hours per week. Diet quality was assessed using a diet score based on recommendations from the dietary recommendations from the American Heart Association 2020 strategic impact goals^21,22^ considering foods including fruits and vegetables; fish and shellfish; sodium; sugar-sweetened beverages; whole grains; nuts, legumes, and seeds; processed meat; and saturated fat. Intake of energy drinks was not counted toward the sugar-sweetened beverage intake criterion of this score.

### Statistical Analysis

Data were analyzed from October 1, 2021, to September 28, 2023. We applied generalized estimating equation (GEE) regression models to examine associations of energy drink intake with odds of APOs separately and combined using odds ratios (ORs). We used a robust correlation structure to account for correlations among repeated observations (pregnancies) contributed by a single participant. Our model adjusted for age (continuous), highest level of education achieved (without a bachelor’s degree, bachelor’s degree, or master’s degree or higher), race and ethnicity (White, racial and ethnic minority group), smoking status (current smoker, noncurrent smoker), parity (yes or no), marital status (never married, ever married or partnered), BMI (<25, 25-30, or >30), physical activity (tertiles), diet quality (tertiles), total energy intake (tertiles), use of multivitamin supplements (yes or no), and total number of years of shift work (0, ≤0.5, 0.6-1.9, 2.0-4.0, or >4.0 [NHS3 only]). For participants with missing values of covariates, a missing indicator was created and used. In the pooled analysis, we combined individual data in NHS3 and GUTS into 1 data set and applied GEE models in the pooled data set. We tested for heterogeneity in associations between cohorts by including interaction terms of energy drink intake and cohort into the model, and *P* for interaction was obtained using a likelihood ratio test comparing models with and without interaction terms. We conducted the analyses using SAS, version 9.4 (SAS Institute Inc) and considered a significance level of 2-sided *P* < .05.

## Results

This study included 7304 pregnancies in 4736 participants with information on prepregnancy energy drink intake and 4559 pregnancies in 4559 participants with information on energy drink intake during pregnancy. The proportion of energy drink consumers who became pregnant during follow-up and were thus included in the study was comparable to that of participants who did not become pregnant during follow-up and were thus excluded from the study (eTable in Supplement 1). Energy drink consumption was low in both cohorts, with 230 of 1691 GUTS participants (14%) and 283 of 3045 NHS3 participants (9%) reporting any intake of energy drinks. The mean (SD) intake among consumers was 0.2 (0.3) servings/day in NHS3 and 0.5 (0.8) servings/day in GUTS. Compared with nonconsumers, participants who consumed energy drinks were more likely to smoke, have obesity, and be more physically active and to have lower educational achievement (Table 1). Participants in NHS3 were more likely to have these characteristics than were those in GUTS.

**Table 1.** Baseline Characteristics by Intake of Energy Drinks in the GUTS and NHS3^a^

Characteristic	GUTS (2011)		NHS3 (module 2)	
	Nonconsumers (n = 1461)	Consumers (n = 230)	Nonconsumers (n = 2762)	Consumers (n = 283)
Energy drink intake, mean (SD), servings/d	0	0.5 (0.8)	0	0.2 (0.3)
Age, mean (SD), y	25.9 (2.8)	24.7 (3.2)	30.2 (4.1)	28.8 (3.8)
Total energy intake, mean (SD), kcal	1778 (589)	1987 (694)	1765 (553)	1774 (485)
Physical activity, mean (SD), h/wk	7.8 (6.8)	9.5 (10.0)	7.0 (5.8)	7.3 (5.4)
Diet quality score, mean (SD)^b^	51.8 (8.7)	50.9 (8.2)	51.5 (8.8)	50.9 (7.6)
Education				
Without a bachelor’s degree	9	11	70	74
Bachelor’s degree	48	62	23	22
Master’s degree or higher	42	27	7	4
Race and ethnicity				
Asian	1	2	2	1
Black	0	0	1	1
Hispanic	1	5	1	1
White	97	92	95	96
Other^c^	1	1	1	1
Smoking status				
Noncurrent smoker	88	64	97	90
Current smoker	12	36	3	10
Body mass index^d^				
<25	76	68	65	55
25-30	16	20	22	26
>30	8	12	13	19
Marriage				
Never married	52	62	25	38
Ever married or partnered	48	38	75	62
Parity				
Yes	14	6	29	23
No	86	94	71	77
Years of shift work				
0	NA	NA	29	30
≤0.5	NA	NA	24	22
0.6-1.9	NA	NA	22	20
2.0-4.0	NA	NA	14	18
>4.0	NA	NA	8	7

Abbreviations: GUTS, Growing Up Today Study; NA, not applicable; NHS3, Nurses’ Health Study 3.

^a^ Data are presented as percentage of participants unless otherwise indicated. All data were standardized to the age distribution of the study population.

^b^ Diet score was derived based on recommendations from the dietary recommendations from the American Heart Association 2020 strategic impact goals^21,22^ and ranges from 0 to 80, with a higher score indicating a healthier diet.

^c^ Includes American Indian and Hawaiian or Pacific Islander individuals.

^d^ Calculated as weight in kilograms divided by height in meters squared.

In GUTS, we documented 3194 eligible pregnancies in 1691 participants during 8 years of follow-up. Mean (SD) maternal age was 25.7 (2.9) years, and mean (SD) prepregnancy BMI was 23.6 (4.4). Of 230 participants who consumed energy drinks (age-adjusted percentages), 2% were Asian; 0%, Black; 5%, Hispanic; 92%, White; and 1%, other. Of 1461 participants who reported not consuming energy drinks, 1% were Asian; 0%, Black; 1%, Hispanic; 97%, White; and 1%, other. Of these pregnancies, 590 (19%) ended in a spontaneous loss; 131 (4%) resulted in a preterm delivery; 112 (4%) were affected by gestational diabetes; 222 (7%) were in women who developed hypertensive disorders, including 135 (4%) with gestational hypertension and 87 (3%) with preeclampsia; and 996 (31%) involved at least 1 of these APOs. The median time between energy drink assessment and pregnancy occurrence was 22 months (range, 0.5-55 months). In NHS3, we documented 4110 eligible pregnancies in 3045 participants. Mean (SD) maternal age was 30.2 (4.1) years, and mean (SD) prepregnancy BMI was 24.7 (5.1). Of 283 participants who consumed energy drinks (age-adjusted percentages), 1% were Asian; 1%, Black; 1%, Hispanic; 96%, White; and 1%, other. Of 2762 participants who reported not consuming energy drinks, 2% were Asian; 1%, Black; 1%, Hispanic; 95%, White; and 1%, other. The frequencies of APOs in NHS3 were 751 (18%) for spontaneous pregnancy loss, 173 (4%) for preterm delivery, 208 (5%) for gestational diabetes, 197 (5%) for gestational hypertension, 123 (3%) for preeclampsia, 320 (8%) for any hypertensive disorders, and 1376 (33%) for any APO. The median time between energy drink assessment and pregnancy occurrence was 25 months (range, 0.1-107 months).

In cohort-specific analyses, there was no association between any prepregnancy intake of energy drinks and any of the APOs evaluated in GUTS (Table 2). However, we observed that higher prepregnancy intake of energy drinks was associated with higher risk of gestational hypertension in NHS3. After adjusting for demographic and lifestyle factors, the OR of developing gestational hypertension was 1.65 (95% CI, 1.04-2.61) for consumers compared with nonconsumers in NHS3. The associations of energy drink intake with outcomes did not differ between cohorts. In pooled analyses, energy drink intake was only associated with gestational hypertension (OR, 1.60; 95% CI, 1.12-2.29). No associations were found for pregnancy loss (OR, 0.89; 95% CI, 0.71-1.11), preterm birth (OR, 1.07; 95% CI, 0.71-1.61), gestational diabetes (OR, 0.89; 95% CI, 0.58-1.35), preeclampsia (OR, 0.73; 95% CI, 0.41-1.30), or the composite APO (OR, 1.05; 95% CI, 0.87-1.26).

**Table 2.** Association of Energy Drink Intake Before Pregnancy With Risk of Adverse Pregnancy Outcomes in the GUTS and NHS3

Outcome	GUTS		NHS3		Pooled	*P* value^a^
	Nonconsumers	Consumers	Nonconsumers	Consumers	Consumers vs nonconsumers	
**Pregnancy loss**						
Cases/observations	540/2858	50/336	693/3748	58/362	NA	NA
Unadjusted OR	1 [Reference]	0.75 (0.55-1.02)	1 [Reference]	0.84 (0.61-1.15)	0.80 (0.64-1.00)	NA
Multivariable-adjusted OR^b^	1 [Reference]	0.79 (0.57-1.10)	1 [Reference]	0.90 (0.65-1.25)	0.89 (0.71-1.11)	.61
**Preterm birth**						
Cases/observations	117/2214	14/249	156/2973	17/293	NA	NA
Unadjusted OR	1 [Reference]	1.07 (0.61-1.87)	1 [Reference]	1.11 (0.65-1.91)	1.09 (0.74-1.61)	NA
Multivariable-adjusted OR^b^	1 [Reference]	1.09 (0.61-1.92)	1 [Reference]	NA	1.07 (0.71-1.61)	.74
**Gestational diabetes**						
Cases/observations	100/2215	12/249	192/2974	16/293	NA	NA
Unadjusted OR	1 [Reference]	1.07 (0.58-1.99)	1 [Reference]	0.84 (0.49-1.42)	0.92 (0.62-1.38)	NA
Multivariable-adjusted OR^b^	1 [Reference]	0.96 (0.49-1.90)	1 [Reference]	NA	0.89 (0.58-1.35)	.82
**Gestational hypertension**						
Cases/observations	116/2215	19/249	164/2974	33/293	NA	NA
Unadjusted OR	1 [Reference]	1.49 (0.88-2.55)	1 [Reference]	2.17 (1.42-3.32)	1.87 (1.34-2.60)	NA
Multivariable-adjusted OR^b^	1 [Reference]	1.32 (0.72-2.41)	1 [Reference]	1.65 (1.04-2.61)	1.60 (1.12-2.29)	.28
**Preeclampsia**						
Cases/observations	83/2215	4/249	112/2974	11/293	NA	NA
Unadjusted OR	1 [Reference]	0.42 (0.15-1.16)	1 [Reference]	1.00 (0.50-1.97)	0.73 (0.41-1.29)	NA
Multivariable-adjusted OR^b^	1 [Reference]	0.43 (0.15-1.26)	1 [Reference]	0.89 (0.45-1.79)	0.73 (0.41-1.30)	.20
**Any hypertensive disorder**						
Cases/observations	199/2215	23/249	276/2974	44/293	NA	NA
Unadjusted OR	1 [Reference]	1.03 (0.64-1.65)	1 [Reference]	1.73 (1.19-2.52)	1.40 (1.05-1.88)	NA
Multivariable-adjusted OR^b^	1 [Reference]	0.94 (0.56-1.57)	1 [Reference]	1.38 (0.93-2.05)	1.27 (0.93-1.72)	.10
**Adverse pregnancy outcomes**						
Cases/observations	901/2754	95/299	1249/3638	127/349	NA	NA
Unadjusted OR	1 [Reference]	0.96 (0.74-1.24)	1 [Reference]	1.09 (0.86-1.39)	1.03 (0.86-1.23)	.36
Multivariable-adjusted OR^b^	1 [Reference]	0.94 (0.72-1.24)	1 [Reference]	1.06 (0.83-1.36)	1.05 (0.87-1.26)	NA

Abbreviations: GUTS, Growing Up Today Study; NA, not applicable; NHS3, Nurses’ Health Study 3; OR, odds ratio.

^a^ *P* for heterogeneity. Heterogeneity between cohorts was assessed by including cohort as a covariate and an interaction term of cohort and energy drink intake in the model.

^b^ Multivariable generalized estimating equation adjusted for age (continuous), highest level of education achieved (without a bachelor’s degree, bachelor’s degree, or master’s degree or higher), race and ethnicity (White, racial or ethnic minority group), smoking status (current smoker, noncurrent smoker), parity (yes or no), marital status (never married, ever married or partnered), body mass index (<25, 25-30, or >30; calculated as weight in kilograms divided by height in meters squared), physical activity (tertiles), diet quality (tertiles), total energy intake (tertiles), use of multivitamin supplements (yes or no), and total years of shift work (0, ≤0.5, 0.6-1.9, 2.0-4.0, or >4.0 [NHS3 only]).

We explored factors that might modify the associations between energy drink intake and APOs and conducted stratified analysis by age, BMI, and time between assessment of energy drink intake and pregnancy occurrence. The associations for prepregnancy energy drink intake and any hypertensive disorders were stronger among older participants (*P* = .02 for interaction for gestational hypertension; *P* = .04 for interaction for any hypertensive disorders) (Table 3).

**Table 3.** Odds Ratios of Adverse Pregnancy Outcomes Comparing Participants Who Did and Did Not Consume Energy Drinks Before Pregnancy^a^

Characteristic	Gestational hypertension			Any hypertensive disorder		
	Cases/observations	OR (95% CI)	*P* value^b^	Cases/observations	OR (95% CI)	*P* value^b^
Age, y						
<28	202/2949	1.27 (0.81-1.97)	NA	326/2949	1.15 (0.79-1.68)	NA
≥28	129/2779	2.46 (1.49-4.06)	.02	215/2779	1.54 (0.92-2.58)	.04
BMI						
<25	141/3932	1.33 (0.75-2.38)	NA	269/3932	1.10 (0.69-1.76)	NA
≥25	185/1707	1.57 (0.97-2.54)	.21	266/1707	1.24 (0.80-1.94)	.29
Time from energy drink intake assessment to pregnancy, mo						
<24	170/2772	1.50 (0.93-2.40)	NA	272/2772	1.31 (0.88-1.97)	NA
≥24	162/2959	1.86 (1.14-3.04)	.28	270/2959	1.28 (0.83-1.96)	.46

Abbreviations: BMI, body mass index (calculated as weight in kilograms divided by height in meters squared); NA, not applicable; OR, odds ratio.

^a^ According to baseline age, BMI, and time of pregnancy occurrence by pooling the Growing Up Today Study and the Nurses’ Health Study 3. Multivariable generalized estimating equation adjusted for age (continuous), highest level of education achieved (without a bachelor’s degree, bachelor’s degree, or master’s degree or higher), race and ethnicity (White, racial or ethnic minority group), smoking status (current smoker, noncurrent smoker), parity (yes or no), marital status (never married, ever married or partnered), BMI (continuous), physical activity (tertiles), diet quality (tertiles), total energy intake (tertiles), use of multivitamin supplements (yes or no), and total years of shift work (0, ≤0.5, 0.6-1.9, 2.0-4.0, or >4.0 [NHS3 only]).

^b^ *P* for interaction.

Next, we assessed the associations of energy drink intake during pregnancy with APOs among 4559 pregnancies in NHS3 participants who reported their diet between 20 and 25 weeks’ gestation. After adjusting for demographic and lifestyle factors, we found no associations of energy drink intake during pregnancy with any APOs (eg, any APO: OR, 0.86; 95% CI, 0.41-1.79) (Table 4).

**Table 4.** Association of Energy Drink Intake During Pregnancy With Risk of Adverse Outcomes in the Nurses’ Health Study 3

Outcome	Energy drink intake during pregnancy	
	Nonconsumers	Consumers
**Preterm birth**		
Cases/observations	272/4512	3/47
Unadjusted OR	1 [Reference]	1.06 (0.33-3.44)
Multivariable-adjusted OR^a^	1 [Reference]	1.13 (0.35-3.60)
**Gestational diabetes**		
Cases/observations	338/4512	2/47
Unadjusted OR	1 [Reference]	0.55 (0.13-2.27)
Multivariable-adjusted OR^a^	1 [Reference]	0.45 (0.11-1.78)
**Gestational hypertension**		
Cases/observations	262/4512	2/47
Unadjusted OR	1 [Reference]	0.72 (0.17-2.99)
Multivariable-adjusted OR^a^	1 [Reference]	0.71 (0.17-3.01)
**Preeclampsia**		
Cases/observations	210/4512	2/47
Unadjusted OR	1 [Reference]	0.91 (0.22-3.78)
Multivariable-adjusted OR^a^	1 [Reference]	0.94 (0.22-3.93)
**Any hypertensive disorder**		
Cases/observations	472/4512	4/47
Unadjusted OR	1 [Reference]	0.80 (0.28-2.23)
Multivariable-adjusted OR^a^	1 [Reference]	0.80 (0.28-2.25)
**Any adverse outcome**		
Cases/observations	958/4512	9/47
Unadjusted OR	1 [Reference]	0.88 (0.42-1.82)
Multivariable-adjusted OR^a^	1 [Reference]	0.86 (0.41-1.79)

Abbreviation: OR, odds ratio.

^a^ Multivariable generalized estimating equation adjusted for age (continuous), highest level of education achieved (without a bachelor’s degree, bachelor’s degree, or master’s degree or higher), race and ethnicity (White, racial or ethnic minority group), smoking status (current smoker, noncurrent smoker), parity (yes or no), marital status (never married, ever married or partnered), body mass index (<25, 25-30, or >30; calculated as weight in kilograms divided by height in meters squared), physical activity (tertiles), diet quality (tertiles), total energy intake (tertiles), use of multivitamin supplements (yes or no), and total years of shift work (0, ≤0.5, 0.6-1.9, 2.0-4.0, or >4.0).

## Discussion

We evaluated the association between energy drink intake before and during pregnancy in 2 populations with moderate (14% in GUTS, 9% in NHS3) use of these beverages. Although intake of these beverages before pregnancy was not associated with any outcomes examined in GUTS, prepregnancy energy drink use was associated with higher risk of gestational hypertension in NHS3. This association persisted in pooled analyses of both cohorts. We also found no evidence that intake of energy drinks during pregnancy was associated with a higher risk of other APOs. Nevertheless, the divergence in findings between the 2 study populations along with the low frequency of energy drink consumption are reasons for caution in the interpretation of these findings.

The association of energy drink consumption with blood pressure has been investigated in multiple studies. Most of these studies used crossover study designs and measured participants’ blood pressure after 0.5 to 5 hours of consuming energy drinks.^6^ A meta-analysis of 15 studies with more than 300 participants found that acute energy drink consumption was associated with increased SBP by 4.4 mm Hg and DBP by 2.7 mm Hg.^6^ Another review showed that ingestion of energy drink with caffeine doses of less than 100 mg was not associated with significant changes in blood pressure, and a higher consumption was associated with a dose-response increase in blood pressure (increases of 3-9 mm Hg for SBP and 2-5 mm Hg for DBP per 100-mg increment in caffeine intake via energy drink).^23^ However, few studies have examined the association of long-term energy drink intake with blood pressure, and our study suggests that habitual prepregnancy energy drink intake might be associated with higher risk of gestational hypertension. Although no statistical heterogeneity in association with gestational hypertension was identified between cohorts, there was a positive association in NHS3 but not in GUTS. The NHS3 and GUTS are different in terms of their sample source, as reflected by the baseline characteristics. Participants in NHS3 were older and had higher mean BMI than those in GUTS, both of which were factors associated with APOs. We found significant modification of associations by age, with greater ORs for energy drink use and risk of hypertensive disorder among older participants. These finding suggest that differences in age between cohorts may account for the discrepancy in findings between NHS3 and GUTS. It is possible that energy drink intake and age have a synergistic effect on gestational hypertension, which should be evaluated in future studies.

### Strengths and Limitations

A major strength of our study is the richness of data collected in NHS3 and GUTS. The NHS3 and GUTS are composed of young women of reproductive age, providing an ideal time frame to investigate factors associated with pregnancy-related outcomes. We inquired about pregnancies that participants had experienced in their life and obtained detailed information on pregnancy end date, pregnancy length, pregnancy outcomes, and pregnancy complications. This allowed us to examine whether energy drink intake was associated with both perinatal outcomes and pregnancy complications. Finally, for prepregnancy energy drink intake, we restricted our analysis to pregnancies that occurred after assessment of energy drink intake to prospectively examine potential associations and conducted stratified analysis by time between assessment of energy drink intake and pregnancy occurrence, which we believe strengthened the scientific rigor of our study.

Several limitations also need to be considered. First, intake of energy drinks was self-reported, and some participants may have misreported their exposure. However, FFQs have been shown to be of high validity and reproducibility for capturing dietary exposures.^24,25,26,27^ Moreover, energy drink consumption was assessed preceding the ascertainment of pregnancy, minimizing the possibility of recall bias. Second, given the low consumption rate of energy drinks in the study population, along with the modest intake levels among those who consumed energy drinks, our study may have had insufficient power to detect some significant associations. Third, as pregnancy-related outcomes were also self-reported, measurement error was inevitable. However, previous studies have shown high sensitivity for gestational age (*r* = 0.84), pregnancy loss (*r* = 0.74), gestational diabetes (*r* = 0.64), gestational hypertension (*r* = 0.65), and preeclampsia (*r* = 0.65) compared with self-reported outcomes in medical records.^16^ Fourth, the NHS3 is composed of predominantly White nurses, which could limit the generalizability of the findings to other racial and ethnic groups. However, as they are trained health professionals, they may have mitigated erroneous reporting and provided higher-quality data than the general population, which enhances the internal validity of the study. Fifth, participants who use energy drinks are more likely to have obesity, lower educational attainment, and high-risk behaviors, including smoking, tanning bed use, binge drinking, marijuana use, substance use disorder, and e-cigarette use.^28^ Cross-sectional studies have reported negative health problems associated with energy drink consumption, including stress, anxiety, and depressive symptoms, lack of sleep, and fatigue.^7,8,9^ Given the potential association of these factors with energy drink use, residual confounding and other sources of bias may be present, and we could not establish a causal relationship between energy drink intake and gestational hypertension. Finally, if energy drinks are associated with the chance of conception, restricting participants with at least 1 pregnancy could result in selection bias. However, the percentage of energy drink consumers were among participants included in our study (with at least 1 pregnancy) and those who were excluded (no pregnancy), indicating that the possibility of selection bias was minimal.

## Conclusions

Our study found that energy drink intake was associated with higher risk of gestational hypertension. Given the low prevalence of energy drink intake in the study population, the results should be interpreted with caution. However, our findings suggest that caution regarding energy drink consumption in reproductive-aged individuals should be exercised and that replication in future studies is needed.

## Notes

Supplement 1. **eFigure 1.** A flow diagram of sample selection in the Growing Up Today Study (GUTS) **eFigure 2.** A flow diagram of sample selection in the Nurses’ Health Study 3 (NHS3) **eTable.** Distributions of energy drink intake among participants included in our study (≥1 pregnancy) and participants who did not experience pregnancy

Supplement 2.
Data Sharing Statement
