# Supplementary material for: Intake of Energy Drinks Before and During Pregnancy and Adverse Pregnancy Outcomes
Source: JAMA Netw Open. 2023 Nov 20;6(11):e2344023. doi: 10.1001/jamanetworkopen.2023.44023 (PMC10660164; doi:10.1001/jamanetworkopen.2023.44023)
Supplement: Supplement 1. — eFigure 1. A flow diagram of sample selection in the Growing Up Today Study (GUTS) eFigure 2. A flow diagram of sample selection in the Nurses’ Health Study 3 (NHS3) eTable. Distributions of energy drink intake among participants included in our study (≥1 pregnancy) and participants who did not experience pregnancy [file jamanetwopen-e2344023-s001.pdf]

Supplemental Online Content

Ding M, Markon AO, Dominic O, et al. Intake of energy drinks before and during pregnancy and adverse pregnancy outcomes. *JAMA Netw Open.* 2023;6(11):e2344023. doi:10.1001/jamanetworkopen.2023.44023

eFigure 1. A flow diagram of sample selection in the Growing Up Today Study (GUTS)

eFigure 2. A flow diagram of sample selection in the Nurses' Health Study 3 (NHS3)

eTable. Distributions of energy drink intake among participants included in our study (≥1 pregnancy) and participants who did not experience pregnancy

This supplemental material has been provided by the authors to give readers additional information about their work.

© 2023 Ding M et al. *JAMA Network Open.*

eFigure 1. A flow diagram of sample selection in the Growing Up Today Study (GUTS).

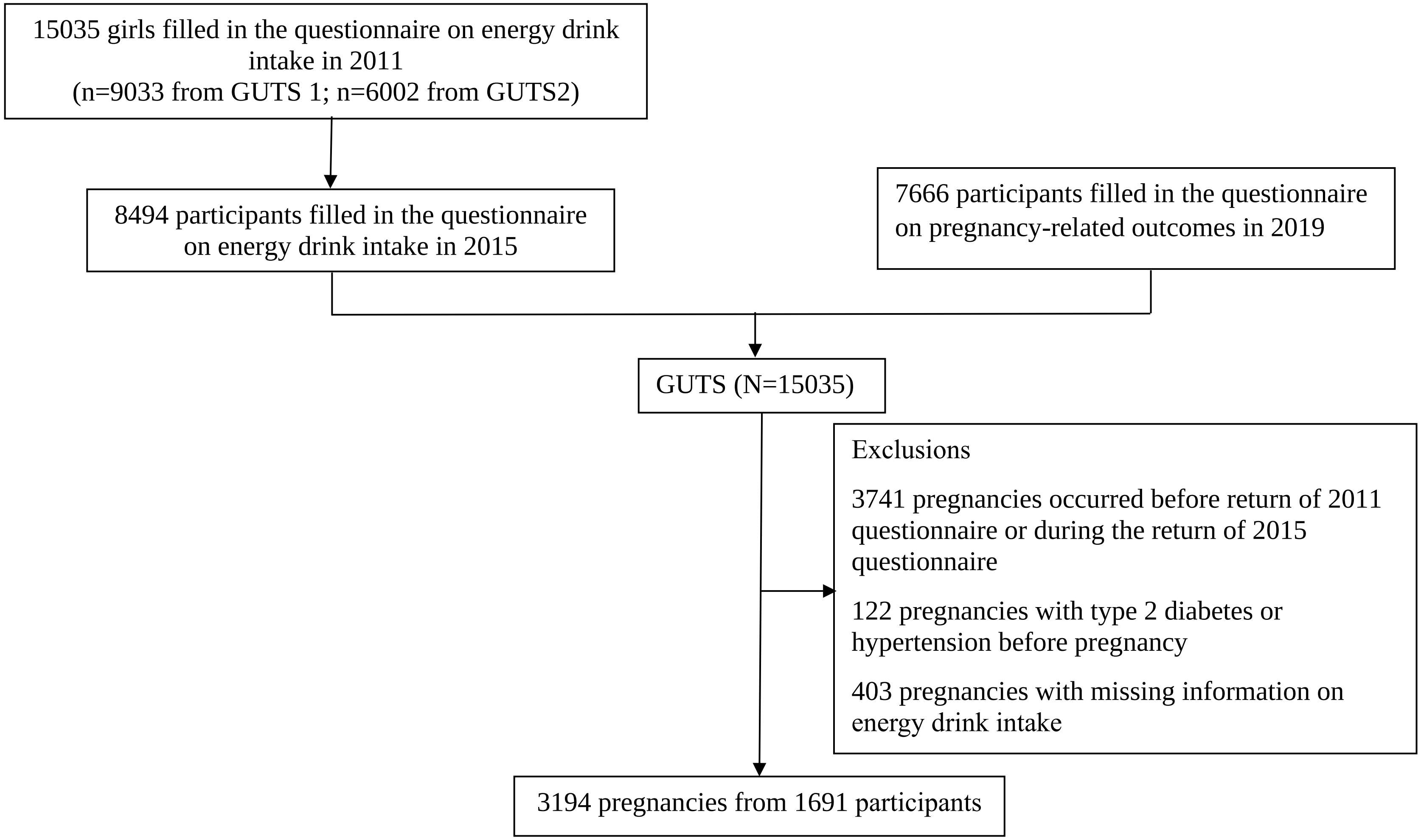

© 2023 Ding M et al. *JAMA Network Open.*

eFigure 2. A flow diagram of sample selection in the Nurses' Health Study 3 (NHS3).

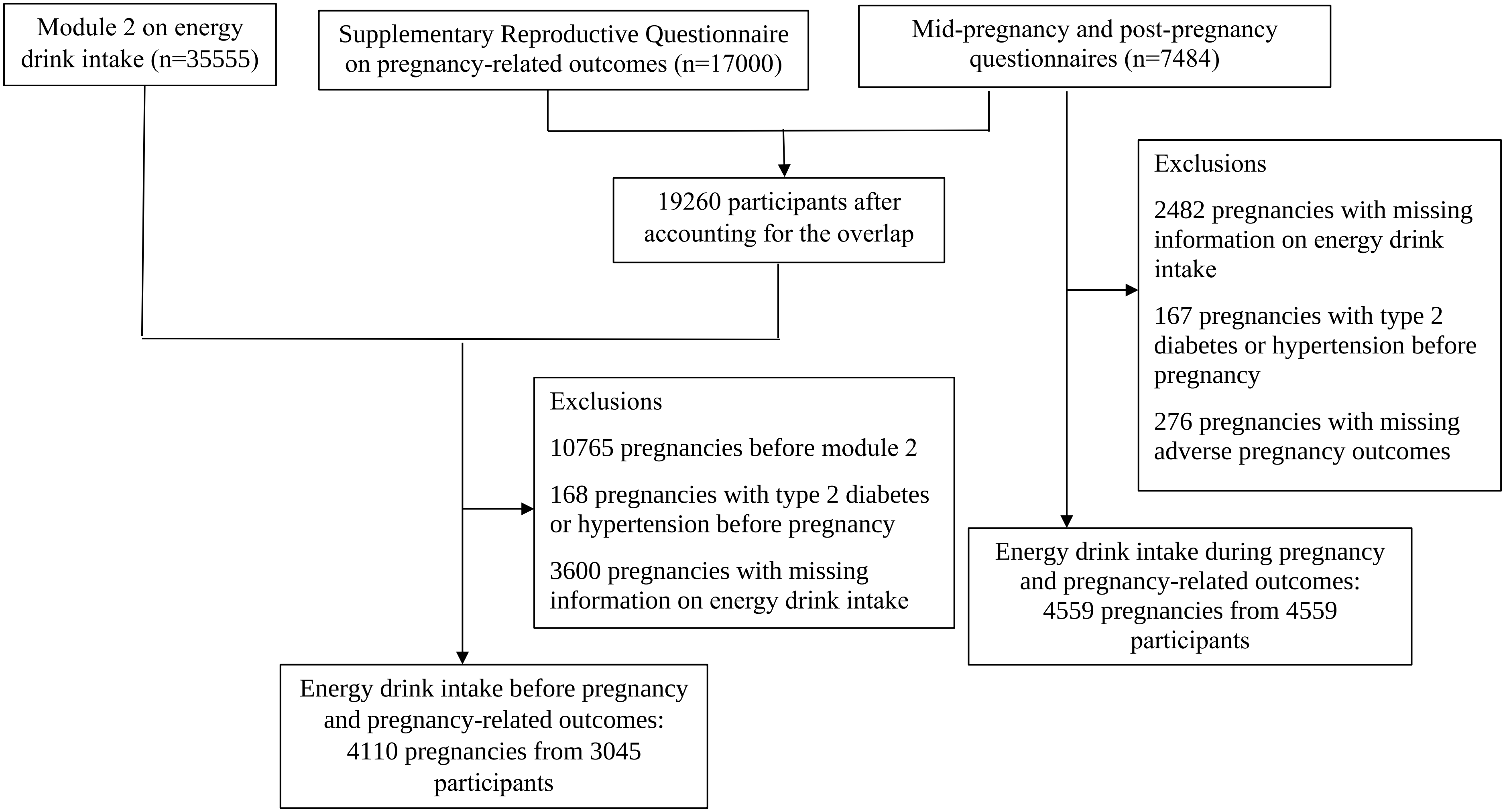

© 2023 Ding M et al. *JAMA Network Open.*

eTable. Distributions of energy drink intake among participants included in our study (≥1 pregnancy) and participants who did not experience pregnancy.

	GUTS		NHS3	
Energy drink intake (servings/day)	≥1 pregnancy, No. (%)	0 pregnancy, No. (%)	≥1 pregnancy, No. (%)	0 pregnancy, No. (%)
0	1461 (86%)	3174 (82%)	2762 (91%)	19259 (89%)
<1	149 (9%)	176 (5%)	266 (9%)	2258 (10%)
1	45 (3%)	325 (8%)	14 (0%)	135 (1%)
≥2	36 (2%)	216 (6%)	3 (0%)	41 (0%)
Total	1691 (100%)	3891 (100%)	3045 (100%)	21693 (100%)

© 2023 Ding M et al. *JAMA Network Open.*
