# Supplementary material for: Intake of Energy Drinks Before and During Pregnancy and Adverse Pregnancy Outcomes
Source: JAMA Netw Open. 2023 Nov 20;6(11):e2344023. doi: 10.1001/jamanetworkopen.2023.44023 (PMC10660164; doi:10.1001/jamanetworkopen.2023.44023)
Supplement: Supplement 2. — Data Sharing Statement [file jamanetwopen-e2344023-s002.pdf]

Data Sharing Statement

Ding. Intake of Energy Drinks Before and During Pregnancy and Adverse Pregnancy Outcomes. *JAMA Netw Open*. Published November 20, 2023. doi:10.1001/jamanetworkopen.2023.44023

Data
Data available: Yes
Data types: Other (please specify)
Additional Information: Access to de-identified data, data dictionaries to code used to generate study data will be granted within the study's data repository system after obtaining authorization from our local IRB and putting in place Data Use Agreements with the parties interested in accessing study data.
How to access data: Access to de-identified data, data dictionaries to code used to generate study data will be granted within study servers after obtaining authorization from our local IRB and putting in place Data Use Agreements with the parties interested in accessing study data.
When available: With publication

Supporting Documents
Document types: Statistical/analytic code
How to access documents: Access to code will be made available within our study's data repository system.
When available: With publication

Additional Information
Who can access the data: Anyone researcher who submits a data request that is approved by our local IRB and whose institution enters into a data use agreement with our institution.
Types of analyses: For the purpose specified in the request only
Mechanisms of data availability: Access through a remote-access data repository with minimal investigator support.
Any additional restrictions: Restrictions defined by institutional data use agreements.
